# 7T MRI for Intracranial Vessel Wall Lesions and Its Associated Neurological Disorders: A Systematic Review

**DOI:** 10.3390/brainsci12050528

**Published:** 2022-04-21

**Authors:** Chen Zhang, Jiong Shi

**Affiliations:** 1 National Clinical Research Center for Neurological Diseases, Beijing Tiantan Hospital, Capital Medical University, Beijing 100070, China; chenzizm@126.com; 2 Department of Neurology, Beijing Tiantan Hospital, Capital Medical University, Beijing 100070, China; 3 Advanced Innovation Center for Human Brain Protection, Capital Medical University, Beijing 100054, China

**Keywords:** 7T MRI, ultra-high resolution, brain, vessel wall, neurological disorders

## Abstract

Intracranial vessel wall lesions are involved in a variety of neurological diseases. The advanced technique 7T MRI provides greater efficacy in the diagnosis of the pathology changes in the vessel wall and helps to identify potential subtle lesions. The purpose of this literature review was to systematically describe and evaluate the existing literature focusing on the use of 7T MRI in the detection and characterization of intracranial vessel wall lesions and their associated neurological disorders, to highlight the current knowledge gaps, and to formulate a framework to guide future applications and investigations. We systematically reviewed the existing articles up to July 2021, seeking the studies that assessed intracranial vessel wall lesions and their associated neurological disorders using 7T MRI. The literature search provided 12 studies that met the inclusion criteria. The most common intracranial vessel wall lesions were changes related to intracranial atherosclerosis (*n* = 8) and aneurysms (*n* = 4), such as intracranial atherosclerosis burden and aneurysm wall enhancement. The associated neurological disorders included aneurysms, ischemic stroke or TIA, small vessel disease, cognitive decline, and extracranial atherosclerosis. No paper studied the use of 7T MRI for investigating vessel wall conditions such as moyamoya disease, small vessel disease, or neurological disorders related to central nervous vasculitis. In conclusion, the novel 7T MRI enables the identification of a wider spectrum of subtle changes and associations. Future research on cerebral vascular diseases other than intracranial atherosclerosis and aneurysms may also benefit from 7T MRI.

## 1. Introduction

Intracranial vessel wall lesions are involved in a variety of neurological diseases, including intracranial atherosclerotic disease (ICAD), aneurysm, arterial dissection, vasculitis, and moyamoya disease. They account for the majority of the intracranial large vessel diseases [1,2], which are closely related to stroke. Characteristics of vessel wall lesions are eccentric wall thickening and enhancement in ICAD [3], enhancement of the aneurysm wall in intracranial aneurysm [4], and concentric enhancement in vasculitis [5], as well as concentric thickening and enhancement without remodeling in moyamoya disease [6]. Information about the specific vessel wall features enables clinicians to better understand the pathology changes, make an accurate diagnosis, and monitor disease prognosis.

Conventional vessel imaging modalities such as magnetic resonance angiography, computed tomography angiography, and digital subtraction angiography provide information about the lumen and show common morphologic changes, e.g., stenosis in the major proximal intracranial arteries, but they fail to assess the vessel wall and to reveal the pathological etiology. Compared with previous neuroimaging methods, vessel wall-magnetic resonance imaging (VW-MRI) is a diagnostic imaging technique that enables specific visualization of the actual intracranial arterial walls. This allows a more direct evaluation and differentiation of the intracranial vasculopathy.

Current technical challenges at 3T VW-MRI are incomplete cerebrospinal fluid (CSF) suppression and limited scan coverage, hindering the full assessment of arterial wall changes [7]. With the advent of more powerful imaging equipment, 7T MRI provides a higher signal-to-noise ratio (SNR), allowing for complete suppression of CSF signal and whole-brain imaging [8]. The SNR increases with the static magnetic field strength (B0), which can be translated into higher spatial resolution and enhanced tissue contrast within clinically reasonable scan times [9], allowing us to obtain more information about abnormalities within the arterial wall, and detect more arterial wall lesions.

As a result, this advanced technique evaluates a wider spectrum of intracranial vascular disease, gives insights into the pathogenesis of intracranial vessel wall lesions, aids the clarification of how vessel wall abnormalities contribute to the onset and progression of neurological disorders, and helps to identify potential subtle lesions.

The purpose of this literature review is to systematically describe and evaluate the existing literature focusing on the use of 7T MRI in the detection and characterization of intracranial vessel wall lesions and their associated neurological disorders, to highlight the current knowledge gaps, and to formulate a framework to guide future applications and investigations.

## 2. Methods

### 2.1. Search Strategy

The systematic review follows the guidelines of the Preferred Reporting Items for Systematic Reviews and Meta-Analyses (PRISMA) statement [10]. We used PubMed to perform the literature search with the following terms: “7T” OR “7 Tesla” OR “seven tesla” OR “ultra-high resolution” AND “MR” OR “MRI” OR “magnetic resonance imaging” AND “vessel wall” OR “arterial wall” OR “artery wall”. Articles published between 1 January 1985 to 26 July 2021 were searched and evaluated.

### 2.2. Eligibility Criteria

Studies were included that investigated 7T MRI for any intracranial vessel wall lesions and their associated neurological disorders. Studies were excluded if they were (1) regarding healthy participants only, (2) regarding no vessel wall lesions associated with neurological disorders, (3) were post-mortem/histology/ex vivo studies, (4) animal studies, (5) MRI sequence/method development, (6) extracranial vessel wall studies, (7) reviews/case series, and (8) with no extractable data.

### 2.3. Study Selection

Studies were first identified by a single researcher via reviewing the title/abstract. Any study that used 7T MRI to investigate the intracranial vessel wall lesions and the related neurological disorders was moved to the next stage of screening. The second round of screening was conducted by two researchers based on the full text, following the eligibility criteria mentioned above. Any discrepancies in judgment were resolved through discussion. Additional studies were also identified by manual reviewing the reference lists of relevant articles.

### 2.4. Data Extraction

The following data were extracted: publication characteristics (first author and year of publication), study design, study population, sample size, 7T MRI protocol for the vessel wall lesions, the characteristics of intracranial vessel wall lesions (lesion type and location), the associated neurological disorders, and the research indications or conclusions.

## 3. Results

### 3.1. Search Results

**Figure 1:**
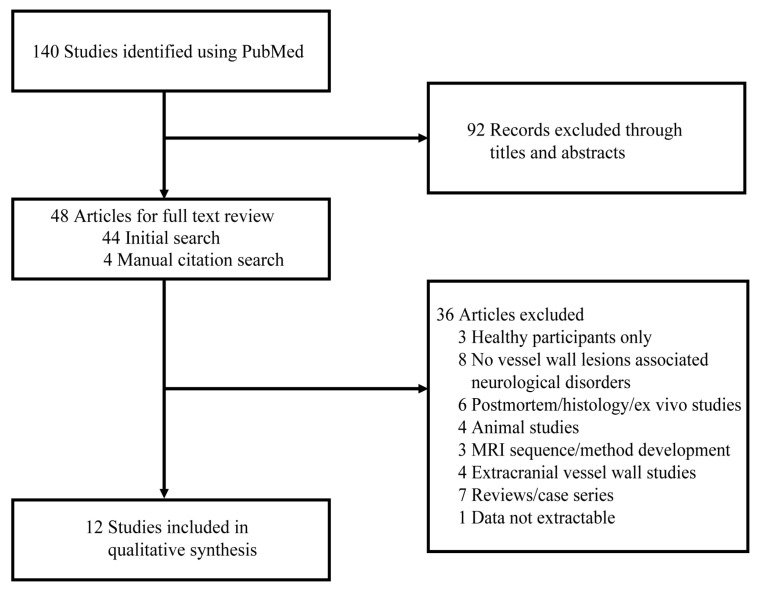
Flow diagram of the study selection. Abbreviations: MRI, magnetic resonance imaging.

The search strategy produced 140 results, among which 44 studies were initially screened by the title/abstract, and 4 additional studies were identified by manual checking the reference lists of the relevant articles, yielding a total of 48 papers for further full-text review. Data extraction for qualitative synthesis was conducted on 12 studies by applying the eligibility criteria and manual citation review (Figure 1).

### 3.2. Study Design and Participants

Most of the studies were published in the recent three years, and only one study was published in 2016. The most common study design was prospective studies (*n* = 7), followed by cross-sectional studies (*n* = 3), and a pilot study (*n* = 1). There was only one longitudinal study investigating the risk of growth or rupture of aneurysms with and without enhancement of aneurysms wall (Table 1).

The number of included participants ranged from 15 to 130. The study population fell broadly into three groups: those with atherosclerosis disease, those with aneurysms, and those with small vessel disease. For patients with atherosclerosis disease, the most common disease was ischemic stroke or transient ischemic stroke (TIA); others included vascular disease and intracranial atherosclerosis disease misdiagnosed as cryptogenic stroke. Studies on aneurysms were on individuals with unruptured intracranial aneurysms. A study on small vessel disease included patients suffering from cerebral lacunar infarcts (Table 1).

#### 3.2.1. 7T MRI Sequences and Intracranial Vessel Wall Lesions Characteristics

Table 1 and Table 2 summarize the 7T MRI image methods and characteristics of intracranial vessel wall lesions, respectively. The common sequences included T1-weighted turbo spin-echo/fast spin-echo, time-of-flight magnetic resonance angiography (TOF-MRA), and pre- and post-contrast enhancement, other less common sequences were T2-weighted images and susceptibility-weighted angiogram. The intracranial vessel wall lesions were classified under ischemia- and hemorrhage-related lesions. All of the affected vessel walls were intracranial major large arteries. For the ischemic-related lesion, the most common type was intracranial atherosclerosis (ICAS), imaging features comprising ICAS burden, location, type, morphology, contrast enhancement, and vessel wall thickness. For the hemorrhage-related lesion, the most common type was aneurysms, and aneurysmal wall enhancement was most commonly evaluated. Additionally, aneurysmal size, number, morphology (fusiform and saccular), focal irregularity changes, or wall shear stress were described.

#### 3.2.2. Vessel Wall Atherosclerosis and Its Related Neurological Disorders

A total of eight 7T MRI vessel wall studies were on investigating intracranial atherosclerosis (ICAS). The 7T MRI image acquisition sequences were similar across the majority of the studies, and T1-weighted fast/turbo spin-echo acquisition sequences were most frequently used. However, there was variability within the literature regarding the selection of key imaging endpoints to assess the atherosclerotic vessel wall lesions, which may reflect a learning curve in image application.

**Figure 2:**
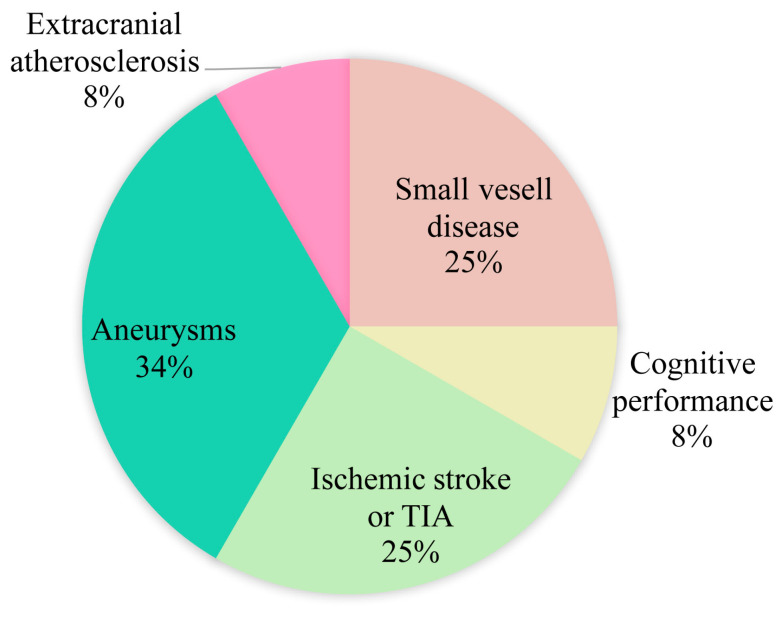
Distribution of studies on intracranial vessel wall lesions related neurological disorders. Abbreviations: TIA, transient ischemic stroke.

Figure 2 shows the distribution of studies on intracranial vessel wall lesions related neurological disorders. The SMART study evaluated ICAS burden with 7T MRI T1 MPIR-TSE in patients with vascular disease, by adding the lesions (defined as focal or diffuse thickening of the vessel wall) per cerebral artery, and found that ICAS burden was associated with cognitive performance [11], markers of extracranial atherosclerosis [12], and cerebral small vessel disease [13]. A 7T MRI study [19] used 3D TOF-MRA and optimized prototype SPACE to investigate the spatial relationship between orifices of lenticulostriate artery and atherosclerosis plaque in patients with lacunar infarcts, showing a decreased number of lenticulostriate artery orifices on the ventral and inferior sides were related with the distribution of middle cerebral artery plaques, suggesting the vulnerability of lenticulostriate artery orifices in ICAS, which may cause lacunar infarcts in the basal ganglia.

Four 7T MRI studies used pre- and post-contrast vessel wall imaging to assess the characteristics of vessel wall atherosclerotic lesions (e.g., concentric or eccentric morphology and contrast enhancement) and their associated disorders. A study investigated the vessel wall enhancement after intra-arterial thrombosuction by using 7T MRI pre- and post-contrast 3D T1-weighted MPIR-TSE and TOF-MRA in patients with ischemic stroke and found patients with intra-arterial treatment had more concentric enhancing foci of the ipsilateral vessel wall, indicating the reactive changes in the vessel wall [17]. The enhancing vessel wall lesions were found to be corresponded with the number of cortical microinfarcts, suggesting the interrelationship between large vessel wall lesion burden and cerebral small vessel disease [18]. Furthermore, patients with macroinfarcts had more concentric and diffuse intracranial atherosclerosis lesions [20]. This specific type of intracranial atherosclerosis lesion may be a marker for a high risk of infarcts. Another study [21] used 7T high-resolution vessel wall imaging to detect atherosclerotic plaques in patients with intracranial atherosclerosis disease initially misdiagnosed as cryptogenic strokes; the results showed that culprit plaques exhibited higher contrast enhancement and concentric configuration, and caused a higher degree of stenosis, highlighting the 7T MRI advantages of identification of intracranial atherosclerosis disease.

#### 3.2.3. Aneurysm Wall Lesions and Their Related Neurological Abnormalities

A total of four 7T MRI vessel wall studies investigating aneurysmal walls were identified. The imaging parameters were varied across the reviewed papers (Table 1). Contrast enhancement was used the most to evaluate the aneurysm instability, formation, and growth. One study [14] reported the parent arteries had increased contrast enhancement in regions closer to the aneurysm’s neck by using 7T high-resolution vessel wall imaging, indicating the localized inflammatory and vasculopathy process in the wall of the parent artery, which may lead to the formation and growth of aneurysms. In a pilot study [16], 7T high-resolution black-blood MRI was used to evaluate the wall enhancement patterns in saccular and fusiform intracranial aneurysms, showing fusiform aneurysms had a higher enhancement and covered a larger surface area than saccular aneurysms, suggesting the differences in vessel wall pathology. There was just one longitudinal study [4] with a median follow-up time of 27 months, which investigated the association between aneurysm wall enhancement and risk of aneurysm instability using 7T T1- weighted sequence, TOF, and time-resolved 3D phase-contrast MR imaging sequences, and the association was significant. The lack of longitudinal study may be due to the high cost of 7T MRI, and it is difficult to accomplish the follow-up in clinical practice. By investigating the role of aneurysmal wall lesions at different time points with a 7T MRI, it is useful to monitor the disease progression and predict the rupture of the aneurysm. Only one study without enhancement was conducted [15], using 7T T1 MPRAGE and T2 TSE 2D images to delineate the wall of the intracranial aneurysm to identify the weak area prone to rupture. At the level of this 7T high-field strength, a hyperintense intravascular signal with a brighter “rim effect” along the vessel wall was observed, and focal irregularities of the aneurysms within this rim effect exhibited higher values of the mean wall shear stress and vorticity, suggesting the altered blood flow parameters within these areas.

No 7T MRI on other intracranial vessel wall lesions such as moyamoya disease, small vessel disease, central nervous vasculitis and dissection, and related neurological disorders were identified. The lack of reports may partly be explained by the novelty of 7T MRI and the relatively small number of individuals affected by the diseases.

## 4. Discussion

This systematic review summarized the use of 7T MRI for the detection and investigation of vessel wall lesions and the associated neurological disorders. The commonly reported vessel wall lesions can be categorized as atherosclerosis and aneurysms wall lesions, which are common etiologies of ischemic stroke and hemorrhagic stroke, respectively. The associated neurological disorders included aneurysms, ischemic stroke or TIA, small vessel disease, cognitive decline, and extracranial atherosclerosis. Other vessel wall lesions conditions such as moyamoya disease, small vessel disease, and central nervous vasculitis were not identified, indicating the novelty of the 7T MRI, which is promising to explore more pathogenesis and detect subtle associated neurological disorders.

Currently, 7T MRI has the highest field strength in approved clinical use in humans, and its application in vessel wall imaging has been shown to be superior to 1.5T and 3T MRI in detecting intracranial vessel wall lesions [22], such as better vessel wall visibility and higher potential to detect lesions. The advantages of 7T over 1.5T and 3T MRI include higher signal-to-noise ratio, increased in-plane resolution, smaller voxels, and stronger susceptibility contrast, which may increase the anomalies conspicuity. Considering the higher capability of identifying abnormalities, and higher diagnostic accuracy, 7T MRI provides new insights into investigating more subtle changes in the vessel wall and its involvement in the pathogenesis of neurological disorders.

### 4.1. Intracranial Atherosclerosis Disease

Intracranial atherosclerosis is a major cause of ischemic stroke [23]. It could lead to atheroma, emboli, and abnormal cerebral blood flow. In this review, it is not surprising to find that intracranial atherosclerosis was the most common disease reported using the 7T VWI MRI technique. However, the wider and various spectrum of the associated neurological disorders identified were noteworthy. For example, intracranial atherosclerosis was associated with cerebral small vessel disease imaging markers, including lacunes, white matter hyperintensity, and cortical microinfarcts. The possible pathway may be the impaired large artery leading to the downstream ischemic lesion. In addition, both post-mortem studies [24,25] and in vivo studies [26,27] confirmed intracranial atherosclerosis was related to cognitive abnormality and dementia. Applying the 7T VWI MRI enabled further evaluation of the premorbid cognitive function and artery-specific relationships. The underlying mechanism is possible due to the strategic brain regions involved with the specific cognitive domains. Apart from that, in this review, we found that intracranial atherosclerosis was related to extracranial atherosclerosis markers, reflecting the advantages of 7T MRI—namely, allowing more potential associations to be fully assessed and found in vivo. By directly visualizing the actual pathology in the vessel wall beyond stenosis, 7T MRI facilitates a more complete and accurate assessment of ICAD, enabling the detection of more subtle changes and relationships. A major limitation of the reviewed atherosclerosis papers is that the evaluation methods of vessel wall lesions remained various, implying the lack of consensus. Future research developing a standardized qualitative and quantitative approach, and a multiparametric scoring system may be useful to accurately and comprehensively measure the vessel wall lesions.

### 4.2. Intracranial Aneurysm

Aneurysm rupture could cause subarachnoid hemorrhage, a subtype of stroke with a poor prognosis [28]. The exact mechanism prompting the formation, growth, and rupture is not fully illuminated. Early identifying the population with a high risk of aneurysm rupture is essential to guide prevention and clinical therapy. One major advantage of investigating intracranial aneurysms at 7T lies in the improved image quality, with higher spatial resolution and signal-to-noise ratio; furthermore, direct visualization of aneurysmal wall and investigation of the associated pathological process by a noninvasive method may be possible. Most of the reviewed articles used ultra-high-field MRI at 7T to delineate the structure, morphology, location, and enhancement features of the wall of intracranial aneurysms, and correlated these vessel walls’ lesional characteristics with blood flow parameters, inflammation, and vasculopathy process, which provides a unique opportunity to estimate the risk of aneurysm development and growth and to better identify novel markers of intracranial aneurysm instability, and will lead to an accurately personalized approach to risk prediction. The limitations of the papers reviewed here are the inconsistent pulse sequence and protocol design. Consensus on defining a minimum clinically achievable 7T imaging quality with appropriate technical parameters and tolerable scan time, as well as attaining good reproducibility and reliability, would be beneficial to generalization.

### 4.3. Moyamoya Disease

No papers studied the use of 7T MRI for moyamoya disease to determine the vessel wall changes, which may be accounted for by the less availability of 7T MRI. Although less common than ICAD, moyamoya disease is particularly important clinically because it is predominant in juvenile patients under 10 years of age [29], causing severe disability due to the development of ischemic stroke [30]. The relatively low proportion of patients may contribute to this deficit. In addition, 7T MRI/MRA has been shown to have higher sensitivity and specificity than 3T MRI/MRA for detecting flow voids in the basal ganglia [31]. Whether 7T MRI is superior to 1.5T/3T MRI for the investigation of vessel wall changes and related neurological disorders remains unclear.

Prior VWI-MRI studies on moyamoya were limited and primarily focused on Asian populations [32,33,34,35]. These studies showed that patients with moyamoya had smaller outer vessel wall diameter and wall thickness, compared with ICAD. One study [36] recruited North American moyamoya patients and applied VWI-MRI to investigate the characterizations in the vessel wall, as well as with moyamoya disease clinical severity. The results showed smaller internal carotid arteries lumen and outer vessel wall diameter in patients with moyamoya, compared with controls, and the diameters decreased with increasing modified Suzuki scores. However, no significant change was detected in vessel wall thickness. Future 7T MRI vessel wall studies with various moyamoya populations on the vessel wall changes may provide more information about the vascular features. In addition, a full evaluation of the characterizations in vessel wall measurements with disease status is necessary to better manage the disease in clinical practice.

### 4.4. Cerebral Small Vessel Disease

Cerebral small vessel disease refers to a series of pathological processes damaging the perforating arterioles, capillaries, and venules [37,38], and it has a crucial role in stroke, dementia, and aging [38]. Though the clinical manifestations and neuroimaging markers were well recognized and studied [39], the exact mechanisms remain incompletely understood and characterized. Conventional neuroimaging markers include white matter hyperintensities, enlarged perivascular spaces, cerebral microbleeds, and lacunes [39]. None of the reviewed articles reported the vessel wall lesions of cerebral small vessel disease at 7T MRI, which is essential to understanding the etiological mechanisms. With advances in MRI vessel wall technology, increasing lines of evidence suggest the role of intracranial vessel wall lesions in the pathogenesis and progression of cerebral small vessel disease. For example, atherosclerotic stenosis with an eccentric plaque and intraplaque hemorrhage of the middle cerebral artery has been noted in CADASIL on magnified T1-weighted imaging [40]. In addition, intramural patchy gadolinium enhancement of the subcortical and leptomeningeal vessels was found in a CADASIL patient who underwent intracranial VWI-MRI [41]. Recent advents in 7T MRI technology may open a new window for scientists to study cerebral small vessel disease, which may help expand the capability to elucidate pathogenesis from the perspective of vessel wall changes.

### 4.5. Central Nervous Vasculitis

Central nervous vasculitis is characterized by vessel wall inflammation, and its diagnosis is challenging and often requires an invasive procedure [42]. VW-MRI promises valuable imaging approaches for a fast and accurate diagnosis. The commonly reported conventional imaging features included vessel wall enhancement and thickening [43] and were shown to have good agreement with histology results [43]. No paper studied the use of 7T MRI for central nervous vasculitis to illuminate the vessel wall lesion and its related neurological disorders, which may be explained by the rare condition that vasculitis affects the central nervous system, and the diagnosis is challenging [44]. Recent case reports showed that 7T MRI could detect the vasculitic changes in the superficial temporal artery and demonstrated superior image quality than 3T [45], and the pathological examination confirmed the 7T MRI results. These lines of evidence suggest that 7T MRI could have a good diagnostic utility for central nervous vasculitis and assist clinical diagnosis and decision making. Moreover, dynamic observations of changes in vessel walls by examination of neuroimaging findings during treatment may aid develop biomarkers, finding more subtle associations, assessing treatment response, and monitoring disease prognosis.

These studies have several main limitations. First, the patient population is largely confined to intracranial atherosclerotic disease and aneurysm, and none of the vessel wall studies use 7T MRI in other conditions such as moyamoya disease, small vessel disease, vasculitis, and dissection. This lack of other investigations may reflect the novelty of 7T MRI vessel wall imaging, its limited availability, and the relatively small number of patients diagnosed with these types of cerebral vascular disease compared with ischemic stroke and aneurysm. Second, the study enrollment is not large, which limits the power of the research. Third, most of them have not conducted longitudinal research to monitor the changes and trajectory of the vessel wall lesions. Fourth, there is a paucity of histological validation of vessel wall lesions. Finally, a lack of consensus on 7T MRI protocols and variability in selecting neuroimaging endpoints may result in differences in detecting neuroimaging features and reproducibility.

Several limitations of the present review need to be acknowledged. By including various study designs for various intracranial vessel wall lesions and their associated neurological disorders, we compiled a heterozygous collection of studies. We did not carry out a risk-of-bias analysis. It is likely that several studies would be susceptible to selective reporting bias. Differences in patient populations, intracranial vessel wall abnormalities, and outcome definitions prevent the performance of a pooled analysis.

Future studies taking a multimodality approach, relating imaging findings to detailed clinical information, biomarkers, and genomics, are warranted to have a better understanding of the pathophysiological mechanisms of vessel wall lesions and the potential correlations. In addition, consensus recommendations of 7T MRI neuroimaging for vessel wall should be made to have a standardized thorough evaluation of the features of the intracranial vessel wall.

## 5. Conclusions

Although not used broadly, 7T MRI shows great potential to identify a wider spectrum of subtle changes and associations. Evidence from intracranial atherosclerosis and aneurysms studies suggests that other cerebral vascular diseases may also benefit from 7T MRI.

## Figures and Tables

**Table 1:** Overview of studies evaluating the vessel wall with 7T-MRI.

Study First Author and Year	Study Design	Major Imaging Methods	No. of Patients	Population
Zwartbol et al. [11], 2020	Cross-sectional study	Philips Healthcare; a 32-channel receive head coil and a volume transmit coil T1 MPIR-TSE; voxel size 0.8 × 0.8 × 0.8 mm^3^ (reconstructed to 0.49 × 0.49 × 0.4 mm^3^); acquisition time 10:40 min	130	Vascular disease
Zwartbol et al. [12], 2019	Cross-sectional study	Philips Healthcare; a 32-channel receive head coil and a volume transmit coil T1 MPIR-TSE; voxel size 0.8 × 0.8 × 0.8 mm^3^ (reconstructed to 0.49 × 0.49 × 0.4 mm^3^); acquisition time 10:40 min	130	Vascular disease
Zwartbol et al. [13], 2021	Cross-sectional study	Philips Healthcare; a 32-channel receive head coil and a volume transmit coil T1 MPIR-TSE; voxel size 0.8 × 0.8 × 0.8 mm^3^ (reconstructed to 0.49 × 0.49 × 0.4 mm^3^); acquisition time 10:40 min	130	Vascular disease
Samaniego et al. [14], 2020	Prospective study	GE MR950 Pre- and post-contrast T1-weighted fast-spin-echo (CUBE), T2-weighted CUBE, and 3D TOF	25	Unruptured intracranial aneurysms
Millesi et al. [15], 2019	Prospective study	Siemens Healthcare; a 32-channel transmit/receive coil T1 MPRAGE; voxel size 0.5 × 0.5 × 0.5 mm^3^; T2 TSE 2D; voxel size 0.4 × 0.4 × 0.4 mm^3^	30	Unruptured intracranial aneurysms
Liu et al. [16], 2020	Pilot study	Siemens Healthcare; a 32-channel receive head coil Pe-contrast and post-contrast black-blood SPACE; voxel size = 0.40 × 0.40 × 0.40 mm^3^; acquisition time 10:29 min 3D TOF-MRA; voxel size = 0.33 × 0.33 × 0.40 mm^3^; acquisition time, 9:16 min	32	Unruptured intracranial aneurysms
Lindenholz et al. [17], 2020	Prospective study	Pre- and post-contrast 3D T1-weighted MPIR-TSE; voxel size 0.8 × 0.8 × 0.8 mm^3^ (reconstructed to 0.49 × 0.49 × 0.49 mm^3^); acquisition time 10:40 min TOF-MRA; voxel size 0.4 × 0.5 × 0.6 mm^3^ (reconstructed to 0.4 × 0.4 × 0.3 mm^3^); acquisition time 9:18 min	49	Ischemic stroke
Lindenholz et al. [18], 2021	Prospective study	Philips Healthcare; either a 16-channel or 32-channel receive coil and a volume transmit-receive coil Pre- and post-contrast T1 MPIR-TSE; voxel size 0.8 × 0.8 × 0.8 mm^3^ (reconstructed to 0.5 × 0.5 × 0.5 mm^3^); acquisition time 10:40 min TOF-MRA; voxel size 0.4 × 0.5 × 0.6 mm^3^ (reconstructed to 0.4 × 0.4 × 0.3 mm^3^); acquisition time 9:18 min	82	Ischemic stroke or TIA
Kong et al. [19], 2019	Prospective study	Siemens Healthcare; a 32-channel receive head coil Optimized prototype SPACE; voxel size = 0.40 × 0.40 × 0.40 mm^3^; acquisition time 10:29 min 3D TOF-MRA; voxel size = 0.23 × 0.23 × 0.36 mm^3^; acquisition time, 7:34 min	15	Lacunar infarcts
Dieleman et al. [20], 2016	Prospective study	Philips Healthcare; a 32-channel receive head coil and a two-channel volume transmit/receive head coil Pre- and post-contrast 3D T1-weighted MPIR-TSE; voxel size 0.8 × 0.8 × 0.8 mm^3^ (reconstructed to 0.4 × 0.4 × 0.4 mm^3^); acquisition time 10:40 min TOF-MRA; voxel size 0.4 × 0.5 × 0.6 mm^3^ (reconstructed to 0.4 × 0.4 × 0.3 mm^3^); acquisition time 9:30 min	18	Ischemic stroke or TIA
Fakih [21], 2020	Prospective study	GE MR950; an 8-channel receive head coil Pre- and post-contrast 3D T1-weighted fast-spin-echo (CUBE); voxel size 0.5 × 0.5 × 0.5 mm^3^; acquisition time 4:27 min 3D TOF; voxel size 0.4 × 0.7 × 0.5 mm^3^; acquisition time 3:49 min T2-weighted CUBE	38	Intracranial atherosclerosis disease misdiagnosed as cryptogenic strokes
Vergouwen [4], 2019	Longitudinal study	Philips Healthcare; a 32-channel receive head coil and a volume transmit coil 3D TFE; voxel size 0.6 × 0.6 × 0.6 mm^3^; acquisition time 4 min T1-weighted sequence, TOF, and time-resolved 3D phase-contrast MR imaging sequence	57	Unruptured intracranial aneurysms

Abbreviations: 3D, three dimensional; MPIR-TSE, magnetization-prepared inversion-recovery turbo spin-echo; MPRAGE, magnetization-prepared rapid acquisition gradient-echo; MRA, magnetic resonance angiography; SPACE, sampling perfection with application-optimized contrasts using different flip-angle evolution; TFE: turbo field echo; TOF, time of flight; TIA, transient ischemic stroke; TOF-MRA, time-of-flight magnetic resonance angiography.

**Table 2:** Characteristics of intracranial vessel wall lesions and the associated neurological disorders.

Study First Author and Year	Vessel Wall Lesions Observation	Vessel Wall Lesions Location	Associated Neurological Disorders	Key Findings
Zwartbol et al. [11], 2020	ICAS burden	Posterior cerebral artery	Cognition: memory and executive functioning	An artery-specific vulnerability (PCA) of memory and executive functioning to ICAS.
Zwartbol et al. [12], 2019	ICAS burden	Circle of Willis and its major branches	Extracranial atherosclerosis: such as carotid intima–media thickness, etc.	Intracranial atherosclerosis was associated with various extracranial markers of atherosclerosis, not supporting a different etiology.
Zwartbol et al. [13], 2021	ICAS burden	Circle of Willis and its major branches	CSVD	Patients with a higher ICAS burden had more CSVD features.
Samaniego [14], 2020	Aneurysmal wall enhancement	Intracranial vessel of aneurysms	Aneurysms: parent vessel enhancement *	A localized inflammatory/vasculopathy process in the wall of the parent artery may lead to aneurysm formation and growth.
Millesi et al. [15], 2019	A brighter “rim effect” along the vessel wall ^#^	Intracranial vessel of aneurysms	Aneurysms: higher values in wall shear stress and vorticity	Focal irregularities unruptured intracranial aneurysms as an indicator for areas of altered blood-flow parameters.
Liu et al. [16], 2020	Aneurysm wall enhancement	Intracranial vessel of aneurysms	Saccular and fusiform aneurysm	Intracranial fusiform aneurysms had enhancement of higher signal intensity and covered a larger surface area than saccular aneurysms, which might indicate differences in vessel wall pathology.
Lindenholz et al. [17], 2020	Vessel wall enhancement after intra-arterial thrombosuction	Intracranial large artery	Ischemic stroke: concentric enhancement on the ipsilateral side with intra-arterial treatment	Patients with intra-arterial treatment had more concentric enhancing foci of the ipsilateral vessel wall, indicating reactive changes in the vessel wall.
Lindenholz et al. [18], 2021	Vessel wall thickening and enhancement	Intracranial large artery	Cerebral parenchymal changes: manifestations of CSVD	Interrelationship between large vessel wall lesion burden and cerebral parenchymal manifestations often linked to CSVD or, alternatively, that vascular changes occur in both large and small intracranial arteries simultaneously.
Kong et al. [19], 2019	Orifices of LSA and the locations of atherosclerotic plaques	MCA	Lacunar infarcts	The vulnerability of LSA orifices in ICAS was supposed to be the cause of lacunar infarcts in basal ganglia.
Dieleman et al. [20], 2016	Location and type of ICAS	Intracranial large artery	Cortical microinfarcts and macroinfarcts	Patients with macroinfarcts had more concentric and diffuse ICAS lesions. This specific type of ICAS lesion may be a marker for ICAS at higher risk of infarcts.
Fakih [21], 2020	Plaque morphology and contrast enhancement, stenosis degree	Intracranial large artery	Intracranial atherosclerosis disease	7T MRI allows identification of intracranial atherosclerosis disease. Culprit plaques had higher CR, caused a higher degree of stenosis, and had concentric morphology.
Vergouwen [4], 2019	Aneurysm wall enhancement	Intracranial vessel of aneurysms	Aneurysm instability	Aneurysm wall enhancement is associated with an increased risk of aneurysm instability.

* Parent vessel enhancement was assessed over regions located 3 and 5 mm from the aneurysm’s neck. ^#^ “Rim effect” indicates a hyperintense intravascular signal on nonenhanced images. Abbreviations: CR, contrast enhancement ratio; CSVD, cerebral small vessel disease; ICAS, intracranial atherosclerosis; LSA, lenticulostriate artery; MCA, middle cerebral artery; PCA, cerebral artery.

## Data Availability

Not applicable.
